# Cancer astrocytes have a more conserved molecular status in long recurrence free survival (RFS) IDH1 wild-type glioblastoma patients: new emerging cancer players

**DOI:** 10.18632/oncotarget.25265

**Published:** 2018-05-08

**Authors:** Sara Franceschi, Francesca Lessi, Paolo Aretini, Valerio Ortenzi, Cristian Scatena, Michele Menicagli, Marco La Ferla, Prospero Civita, Katia Zavaglia, Claudia Scopelliti, Alessandro Apollo, Francesco Giovanni Carbone, Riccardo Vannozzi, Generoso Bevilacqua, Francesco Pasqualetti, Antonio Giuseppe Naccarato, Chiara Maria Mazzanti

**Affiliations:** ^1^ Fondazione Pisana per la Scienza, Onlus, Pisa, Italy; ^2^ Department of Translational Research and New Technologies in Medicine, University Hospital of Pisa, Pisa, Italy; ^3^ SOD Molecular Genetics, University Hospital of Pisa, Pisa, Italy; ^4^ Tumor Cell Biology Unit, Istituto Tumori Toscana, Florence, Italy; ^5^ Neurosurgery Department, University Hospital of Pisa, Pisa, Italy; ^6^ Pathological Anatomy Section, San Rossore Clinic, Pisa, Italy; ^7^ Radiotherapy Department, University Hospital of Pisa, Pisa, Italy

**Keywords:** glioblastoma, next generation sequencing (NGS), recurrence free survival (RFS), onco-drivers, tumor suppressors

## Abstract

Glioblastoma is a devastating disease that despite all the information gathered so far, its optimal management remains elusive due to the absence of validated targets from clinical studies. A better clarification of the molecular mechanisms is needed. In this study, having access to IDH1 wild-type glioblastoma of patients with exceptionally long recurrence free survival (RFS), we decided to compare their mutational and gene expression profile to groups of IDH1 wild-type glioblastoma of patients with shorter RFS, by using NGS technology. The exome analysis revealed that Long-RFS tumors have a lower mutational rate compared to the other groups. A total of 158 genes were found differentially expressed among the groups, 112 of which distinguished the two RFS extreme groups. Overall, the exome data suggests that shorter RFS tumors could be, chronologically, in a more advanced state in the muli-step tumor process of sequential accumulation of mutations. New players in this kind of cancer emerge from the analysis, confirmed at the RNA/DNA level, identifying, therefore, possible oncodrivers or tumor suppressor genes.

## INTRODUCTION

Glioblastoma multiforme (GBM) is a devastating brain cancer that can result from *ex-novo* or proceed from a lower-grade astrocytoma. It is the most aggressive and lethal brain tumor in humans classified as Grade IV astrocytoma. The incidence of glioblastoma is of 2 or 3 cases in 100,000 people in Europe and North America accounting for 52% of all human primary brain tumors [1]. Present treatment approaches for glioblastoma comprehend surgical resection, radiation therapy and chemotherapy. Unfortunately in spite of aggressive treatments, patients’ response is poor and average survival is 15 months after diagnosis [2, 3]. An optimal management of patients with glioblastoma is still elusive because of the lack of data validated by clinical trials and of the great heterogeneity and fragility of this patients’ population in terms of physical condition, co-morbidity state, tolerance treatment and clinical prognosis [4]. Thus to increase the survival of patients with glioblastoma the development of novel therapies is clearly needed. To advance further the currently available therapies for glioblastoma, new treatment approaches are being explored aiming to improve survival rates.

This study had the intent to provide novel information on glioblastoma tumor aggressive behavior by investigating with deep sequencing the gene mutational and expression status of glioblastoma tumors with different recurrence free survival time after first diagnosis. A refined selection was achieved to obtain a highly homogeneous IDH1 wild-type GBM patient cohort divided among three groups with primary glioblastoma but with different recurrence free survival time (RFS) such as: 6 Short (S) less than 6 months, 3 Medium (M) between 16 and 23 months and 4 Long (L) over 25 months. Finding out why a patient with glioblastoma survives longer compared to a patient with the same diagnosis may lead to: 1) identify a genetic landscape that can be used to give more specific prognosis and hopes to these terminal patients, 2) develop therapeutic strategies that target the molecular pathways characteristic and responsible for a major or minor aggressiveness.

## RESULTS

### Demographic characteristics of patients

The L group presented an average age of 53 years. The M group an average age of 58 years and the S group of 56. The gender distribution was 8/13(61%) females and 5/13 (49%) males. Each patient was provided with various molecular diagnostic results such as: EGFR-ampl, EGFR- variant III presence, MGMT methylation status, IDH1-R132 and IDH2-R172 molecular status (Table 1). The comparison between the patient’s characteristics, within each group, to the length of RFS did not identify any statistical significant association (data not shown). Furthermore, no statistical significant correlations were observed among the molecular alterations provided at the diagnosis and RFS (data not shown).

**Table 1:** Selected cases for WES and WTS analysis: demographic characteristics of the patient population at the time of diagnosis, and molecular characterization of the glioblastoma tumors

RFS Month	RFS ID	ID Sample	Age	Sex	Tumor Size (cm^2^)	Brain Region	EGFR	EGFRvIII	MGMT	IDH1/2	RNAseq (WTS)	Exome Seq (WES)
2	S	A	58	F	70	Frontal	AMPL	Trunc.	MET	WT	X	X
3	S	B	60	F	52	Parietal	Not AMPL	WT	UNMET	WT	X	X
3	S	C	58	F	33	Right- temporal	AMPL	WT	MET	WT	X	X
5	S	D	62	M	7	Right-insula	Not AMPL	WT	UNMET	WT	X	
5	S	E	70	M	28	Left-prerolandic (frontal)	Not AMPL	WT	MET	WT	X	X
5	S	F	58	F	146	Right frontal	Not AMPL	WT	UNMET	WT	X	
16	M	G	58	M	90	ND	AMPL	WT	MET	WT	X	
18	M	H	56	M	13	ND	Not AMPL	WT	UNMET	WT	X	
23	M	I	60	F	48	Basal temporal lobe	AMPL	Trunc.	MET	WT	X	
25	L	J	61	M	17	ND	Not AMPL	WT	UNMET	WT	X	X
30	L	K	57	F	33	Frontal	AMPL	WT	MET	WT	X	X
32	L	L	44	F	3	Frontal	AMPL	Trunc.	MET	WT	X	X
42	L	M	50	F	16	ND	ND	WT	UNMET	WT	X	X

RFS, recurrence-free survival; S, short group; M, medium group; L, long group; letters from A to M represents each ID samples; WES, whole exome sequencing; WTS, whole transcriptome sequencing; EGFR, epidermal growth factor receptor; EGFRvIII, EGFR variant III; MGMT, O(6)methylguanine-DNA methyltransferase; IDH1, isocitrate dehydrogenase1; MET, methylation; UNMET, no methylation; Trunc, presence of truncated form; AMPL, amplified; WT, wildtype; ND, not determined.

### NGS sequencing results

#### Exome analysis

#### Total number of variations

**Figure 1:**
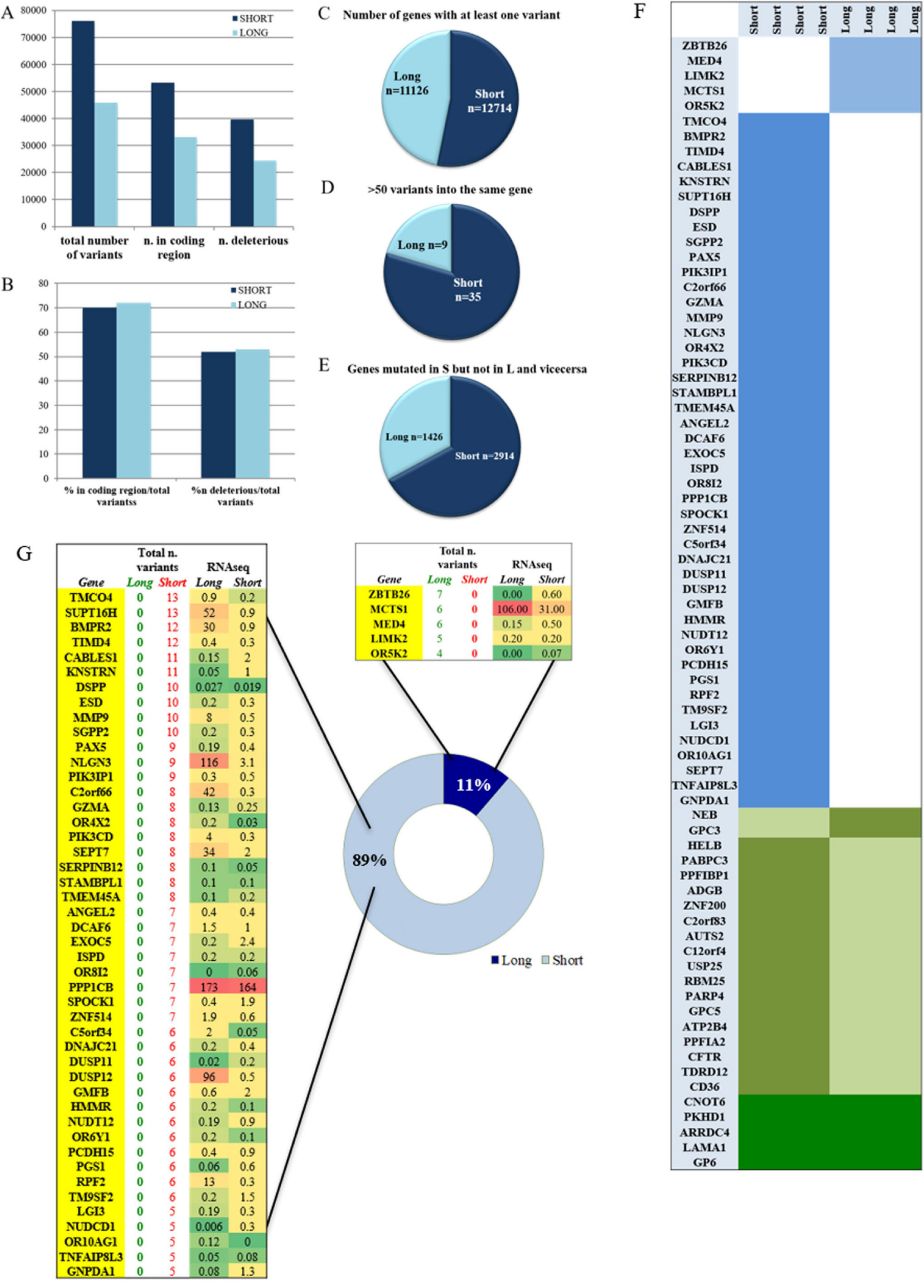
Mutational analysis of S and L samples (**A**) total number of variants in the S and L groups divided in coding sequence variants and deleterious. (**B**) percentage of coding region and deleterious variants in the total number per group. (**C**) number of genes that were carrying at least one variant. (**D**) hyper-mutational gene status defined by presence of more than 50 variants per gene. (**E**) number of genes that were exclusively mutated in one group and wild type in the other. (**F**) list of mutated genes shared by all samples of the same group (light blue) and not mutated in the other, list of mutated genes in both groups but with identical variants shared by one group and not the other (olive green), and identical gene variants shared by all samples of both groups (dark green). (**G**) list of genes that are carrying more than four variants per groups and at least one variant in each sample of the same group. The L group had 5 genes always mutated in each sample compared to the 46 genes of the S group. The total number of variants found per gene is also reported in each group. The last two columns of the two tables show the expression values obtained by RNAseq analysis. RNAseq expression value levels are reported in fragments per kilobase million (FPKM). Levels of expression are also shown with a green/yellow/red color scale of the differentially expressed genes (DEG): dark red indicates the highest expression level and dark green the lowest.

8 GBM tissues belonging to the two extreme groups S (*n* = 4) and L (*n* = 4) were subjected to whole exome sequencing (WES). The number of mutated gene was 15610 while the overall number of molecular alterations, coding sequence region variations and deleterious variations was respectively 76170, 53319, 39609 in the S group and 45903, 33050 and 24328 in the L group as shown in Figure 1A. Between the S and L group, despite the high difference in the total number of variations, the percentages of coding sequence and deleterious variations over the total, and of deleterious variation over the coding sequence, were very similar as shown in Figure 1B.

The S group carried at least one variant in 12714 genes, while the L group in 11126 genes with a statistical significant difference (*P* < 0.0001) as shown in Figure 1C.

Overall, a hyper mutational status was observed significantly higher in the S group where 35/12714 genes contained more than 50 variants compared to 9/11126 in the L group with a *P* = 0.0005 (Figure 1D). The number of genes that were exclusively carrying a variant in the S group but not in the L group was 2914 compared to 1426 genes in the L group with a statistical significant *P* ≤ 0.0001 (Figure 1E).

#### Mutated genes associated with the S and/or L group

**Figure 2:**
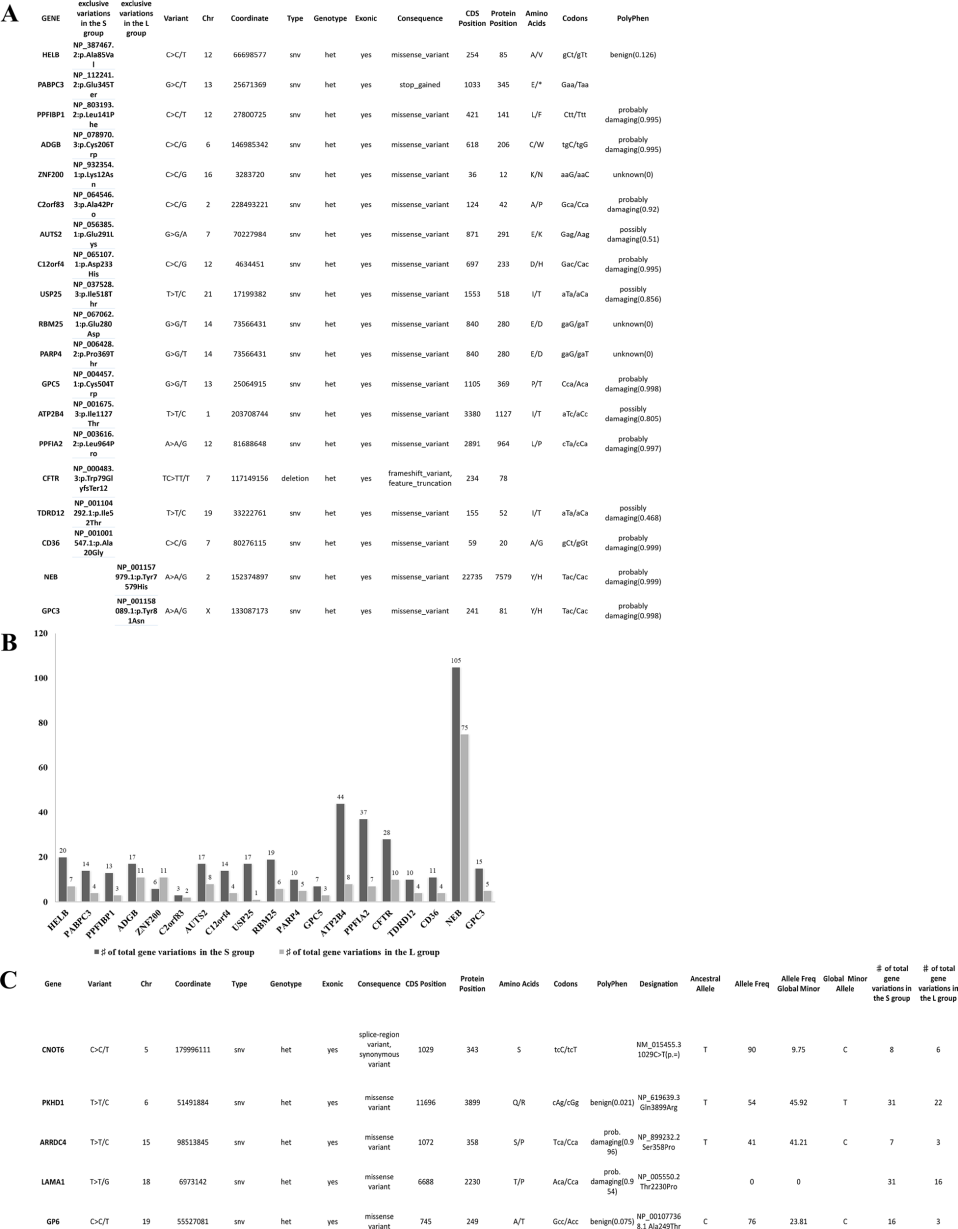
Details of variants per group and hypermutational status (**A**) 19 identical unique variants exclusively shared by all samples in the S or L group, 17 and 2 respectively. All variants were SNV except for deletion in the CFTR gene. 18 variants were of missense type and 1 was a stop-gained type in the PABPC3 gene. All variants were in heterozygous status. (**B**) the hyper-mutational status of each gene is described in this graph, the S group have all genes with more mutations than the L group except for the ZNF200 gene. The NEB was the most mutated gene with 105 variants in the S group and 75 in the L group. (**C**) all 8 GBM samples share 5 different variants. All variants were SNV in a heterozygous status. 4/5 were aminoacid substitutions. The evaluation of pathogenicity was performed with the PolyPhen tool. The variant allele frequency (minor) had a range between 9% and 45,9% in the total population. Other variants are present in the same genes but not shared by all samples, as shown in the last column of the table.

A thorough analysis identified a series of variants or mutated genes, that selectively happened to be present in both groups or in just each of the two. Filtering was strict and done accordingly, based mostly on investigating identical events occurring in all samples within the identicle group. As shown in Figure 1F, the S and the L groups are distinct by specific mutated genes and by specific variants (Supplementary Figure 1), as well as they share same variants and mutated genes (Figure 2A). It is clear from Figure 1F how the S group maintains its higher mutational rate. In details:

#### Mutated genes shared by S samples or L samples exclusively

We focused on the genes with more than 4 variants described per group shared by all samples in the same group. As shown in Figure 1G, the S group had 46 genes mutated in all samples compared to only 5 genes in the L group indicating again a higher mutational rate within the S group. The genes that resulted mutated in each sample of the same group are reported together with the total number of variations/gene and the RNAseq gene expression value (Figure 1G). We focused our attention purely on the differentially expressed genes (DEG) in the S and L group.

#### L group

In the L group only MCTS1 resulted up-regulated compared to the S group, albeit without statistical significance. Details about the variant type per gene will be found in Supplementary Figure 1.

#### S group

8 genes resulted having a down-regulation in the S group compared to the L group: SUPT16H, BMPR2, MMP9, NLGN3, C2orf66, SEPT7, DUSP12 and RPF2. The remaining 38 genes presented a very low RNA expression value for both groups (Figure 1G). BMPR2, MMP9, RFP2 and SEPT7 are described as tumor suppressor genes (TSG) [5–8]. Of the eight genes, the down regulation of 4 was statistically significant: BMPR2 with a *P* = 0.02, MMP9 with a *P* = 0.05, NLGN3 with *P* = 0.003 and SEPT7 with *P* = 0.009 (Supplementary Table 1). All 8 genes were hit by distinct kind of mutations such as single nucleotide variations, deletions and insertions, with different degrees of damaging effect as shown in Supplementary Figure 1. It is important to point out that MMP9 and SEPT7 were carrying a deletion variant with a feature of a truncation in each GBM sample (A, B, C, E, Supplementary Figure 1) implying a disruption of the gene with very likely loss of gene expression, as we indeed observe in the RNAseq data (Figure 1G). Interestingly, to point out that SEPT7 was identified, recently, as a known tumor-suppressor gene that inhibits glioblastoma cell migration and invasion, as a direct target of miR-127-3p [8].

#### Same variants 100% exclusive of S or L

We identified a series of identical variants exclusively present in one group and shared by all samples in the same group. As shown in Figure 2A, 17 variants were shared identically by all samples in the S group but not present in L group, while only 2 variants were shared by all samples in the L group. It is important to point out that all S GBM samples carry in the PABPC3 gene a stop codon gained variant, which has been already reported in the dbSNP of the NCBI database. The PABPC3 gene product binds the poly(A) tail of mRNA and maybe involved in cytoplasmic regulatory processes of mRNA metabolism [9]. There are no reports on the role of PABPC3 in cancer, and this is the first time that an association to cancer is described. The hyper-mutational status of the total 19 genes of the two groups is described in Figure 2B.

#### Variants shared 100% by all samples (S and L)

We identified 5 different variants that were shared by all 8 samples in the following genes: CNOT6, PKHD1, ARRDC4, LAMA1 and GP6. Each variant was a single nucleotide variation (SNV). In Figure 2C, there is a description of each variant with details of location, genotype, evaluation of pathogenicity, allele frequency and mutational status of the gene. The expression levels are very similar between sample groups (Supplementary Table 1) and only the PKHD1 and LAMA1 genes have been implicated, so far, in cancer [10, 11]. A number of other variants were present in the same five genes, but not shared by all samples, as shown in the last column within the Table.

### Transcriptome analysis

#### Number of differentially expressed genes (DEGs) among S, M, and L groups

**Figure 3:**
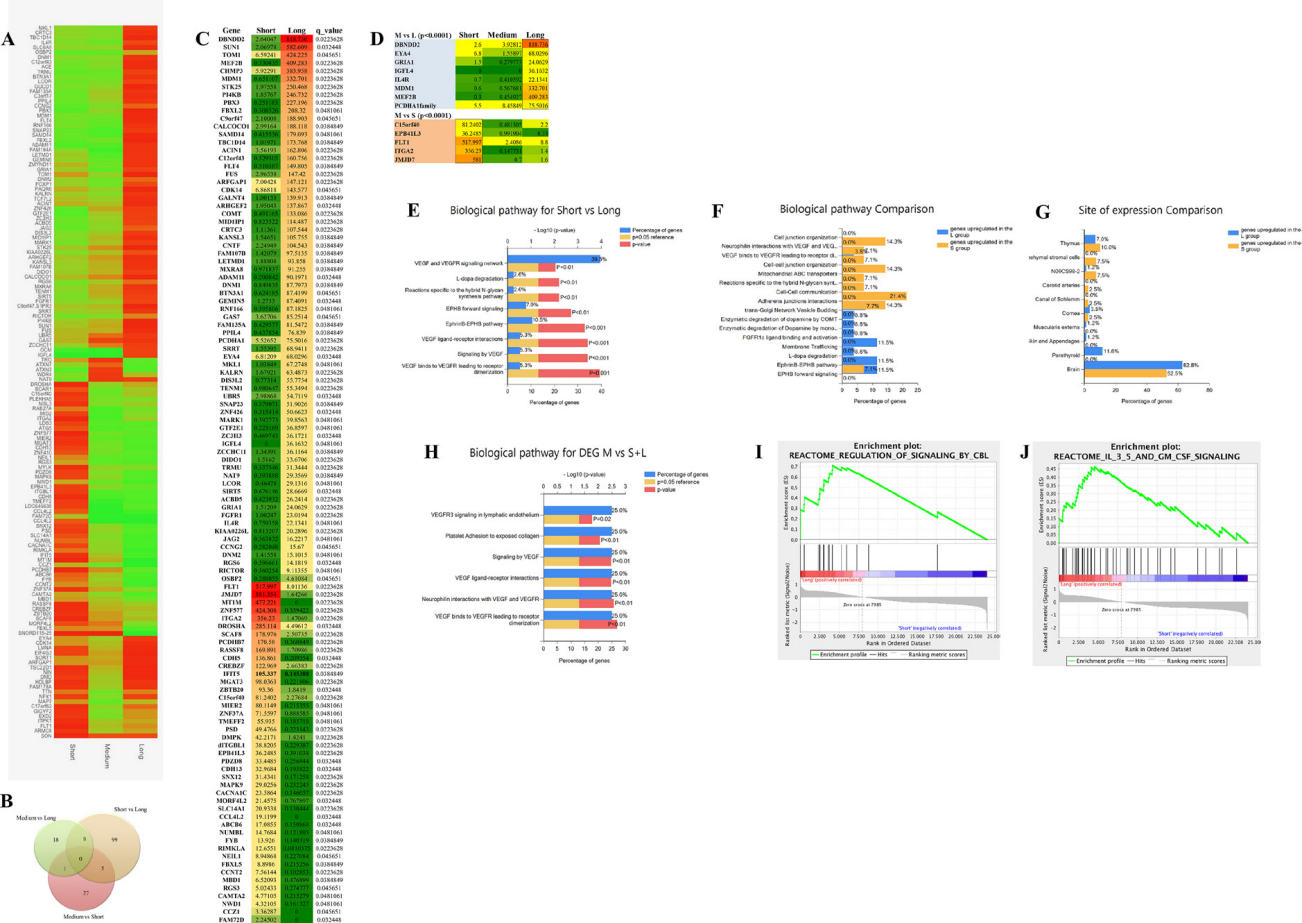
Transcriptome data analyses (**A**) heatmap of 158 statistically significant differential expressed genes (DEG) among the S, M and L group. Levels of expression are shown with a green/red color scale, red indicating over-expression and green down-expression (**B**) Vent diagram reporting the distribution of the 158 DEG through the different groups comparison. (**C**) list of 112 statistically significant DEG between the S and L group representing the two extreme groups of the RFS time range. RNAseq expression value levels are reported in fragments per kilobase million (FPKM). Levels of expression are also shown with a green/yellow/red color scale of the differentially expressed genes (DEG): dark red indicates the highest expression level and dark green the lowest. The false discovery rate (FDR) is below 0.05 (*P* < 0.0002). (**D**) list of 13 of the 60 statistically significant DEGs between the M group and the L group (*n* = 8) and the S group (*n* = 5), that are in common with the results of the S and L statistical comparison analysis. The false discovery rate (FDR) is below 0.05 (*P* < 0.0002). (**E**) Funrich enrichment analysis (FEA) of biological pathways, based on the 112 significant genes distinguishing the S and L group. (**F**) FEA of biological pathways comparing S to L group. (**G**) FEA comparison of site of expression. (**H**) FEA based on 13 significant genes differentially expressed between the M and the S and L group and in common with the S-L statistical comparison. (**I**, **J**) gene enrichment analysis (GEA), based on whole transcriptome dataset, comparing the S and L groups revealed two gene sets involved in CBL signalling (I) and IL3, 5 and GM CSF signalling (J) at a false discovery rate of 0.9% and 3.7% respectively, *P* < 0.01.

An unsupervised variable selection was performed by choosing only genes with a false discovery rate (FDR) below 0.05 (*P* < 0.0002). We could identify 158 genes differentially expressed among the S, M and L groups (Figure 3A). A heatmap of the 158 significant genes shows how the M group carries similarity shared alternatively with the S and L group (Figure 3A). In the Venn diagram, 112 genes represent the highest number of statistically significant DEGs that defines the difference between the S and L groups, which are at the opposite extremities of the RFS time range (Figure 3B, 3C), Several of genes have been identified with an extensive thorough documentation on cancer involvement (Figure 3C). Some of these genes belong to the same family and resulted to be significantly deregulated such as FLT1 and FLT4, CDH5 and CDH13, ITGA2 and ITGBL1, FBXL2 and FBXL5, PCDHA1 and PCDHB7, and RGS3 and RGS6 genes [12–20]. It is noteworthy that MT1M, MAPK9 and RGS3 are up-regulated in the more aggressive group S as expected from the literature [17, 21, 22]. Potential tumor suppressor genes GAS7, RGS6, ADAM11 and FBXL2 are up-regulated in the lesser aggressive group L [16, 23, 24]. It is important to pinpoint that among the oncogenes upregulated in the S group, we identified the MT1M gene, which has been already reported associated to a more aggressive glioma behavior. Metallothioneins (MTs) are intracellular heavy metal binding proteins and recent literature suggests that they are involved in key mechanisms associated with longevity. Elevated levels have also been shown to enhance the migration and invasion of human glioma cells. Authors validated this result by analysing the expression data of 210 GBM patients taken from the TCGA database. They show that a subset of GB patients with high levels of MTs has decreased survival [21].

We noticed also the GRIA1 and RIMKLA genes, which belong to the gene system controlling the glutamergic pathways. Glutamate is the major excitatory neurotransmitter in the nervous system and appears to play a central role in the malignant phenotype of glioblastoma via multiple mechanisms [25, 26].

Interestingly a novel gene, IFIT5, an interferon induced protein, is highly expressed within the S group. No data of IFIT5 involvement in cancer has been reported so far. However, the Human Protein Atlas (www.proteinatlas.org/ENSG00000152778-IFIT5/cancer) shows several cases of only glioma exhibiting cytoplasmic strong positivity. The remaining malignant tissues are all completely negative. IFIT5 is expressed in normal glial cells only, and gets upregulated in glioma behaving like a glia cell marker as GFAP. It is important to investigate in better details the involvement of IFIT5 in glioblastoma development and if it exists an association with survival/aggressiveness.

Of the 60 DEGs, in the M group compared to S and L, 13 genes are in common with the S and L statistical comparison analysis, and may represent the genes that are the most important in diverging the S from the L group in our case study (Figure 3D) (Supplementary Figure 2).

### Molecular pathway analysis by Fun RICH

To be able to give a meaning to the dozen of deregulated DEGs, we used a stand-alone software (FunRich) tool used mainly for functional enrichment and interaction network analysis of genes in order to find meaningful results for biological pathway investigation. As shown in Figure 3E, the VEGFR, Ephrine and Dopa degradation pathways are mostly involved in the difference between the less aggressive group of patients (L) compared to the more aggressive one (S). Performing the analysis comparing the two groups one to another we identify that the vascular endothelial growth factor (VEGF), cell-cell communication and adherent junctions interactions pathways are more represented and expressed within the S group (Figure 3F). Multiple strategies have been developed to target VEGF/VEGF receptor (VEGFR)–mediated angiogenesis [27]. The L group is, instead, particularly enriched with genes involved in the Ephrine receptor forward signaling and enzymatic dopamine degradation pathways (Figure 3F). Emerging evidence has indicated that signaling molecules, previously implicated in axon guidance, are important regulators of multistep tumor initiation and progression, these include ephrine. Recent data suggest a model in which ephrin-induced Eph receptor forward signaling inhibits tumor cells proliferation [28]. On the same track, it has been recently described that neurotransmitter receptor signaling genes are the main factors required for glioblastoma growth *in vitro* [29]. The same authors subsequently demonstrated that one of these pathways, mediated by the dopamine receptor subtype 2 (DRD2), plays a critical role in glioblastoma mitogenic signaling and that DRD2 antagonists, clinically used as anti-psychotic drugs, harbor anti-glioblastoma activities [29–31]. Therefore, as shown in Figure 3F, the fact that an enzymatic dopamine degradation pathway is enriched in the L group could account for the less aggressive behavior of this kind of glioblastoma.

Figure 3G shows that the organ sites of expression of the DEGs in the S and L group is mostly represented by the brain, 58.5% and 62.8% respectively. The deregulation of the VEGF/VEGFR pathway was confirmed also analyzing the genes that are differentially expressed between the M group and the S/L groups, and represents the most representative of the divergence between the two extremities of the RFS time range (Figure 3H).

### Molecular pathway analysis by gene set enrichment analysis (GSEA)

A more elaborate enrichment analysis algorithm, was also used to analyze our entire transcriptome data. GSEA is a computational method that determines whether an a priori defined set of genes shows statistically significant, concordant differences between two phenotypes. It is important to stress the fact that the GSEA analysis report highlights enrichment gene sets with a false discovery rate (FDR) of less than 25% as those most likely to generate interesting hypotheses and drive further research. However, in our case having a small number of samples, we used a more stringent FDR cutoff, such as 5%. Two sets of genes turned out to be highly significantly upregulated in the differentiation within the L group from the S group (Figure 3I, 3J) such as the CBL signaling pathway and the IL3, 5 and GM CSF signaling, with a false discovery rate of less than 0.9% and 3.7% respectively. Cbl proteins controls signaling cell-surface receptors ubiquitination, which is a key mechanism regulating the availability of these receptors to interact with extracellular ligands. Malignant cells utilize modified ubiquitination of signaling receptors to augment or attenuate signaling pathways on the basis of whether the outcome of this signaling is conducive or not for tumor growth and survival. Alterations in receptors ubiquitination and degradation are often encountered in cancers. Diverse strategies that enable this evolution often include alterations in ubiquitination and degradation of signaling receptors, such as EGFR receptor [32], which is highly expressed in glioblastoma tissues. This result might indicate that the L tumors are provided by a mechanism of disruption of the EGFR oncogenic activity. In Figure 3J, it is shown the second gene set that resulted significantly upregulated in the L group, which includes genes related to the IL3, 5 and GM CSF signaling. High-affinity binding of GM-CSF, interleukin 3 (IL-3), and IL-5 to their receptors induces a number of key events at the cell surface and within the cytoplasm that are necessary for receptor activation. Multiple biological responses such as proliferation, survival, and differentiation can be transduced from activated GM-CSF, IL-3, or IL-5 receptors [33]. The IL3, IL5 and GM CSF signaling system is basically involved in the enhancement of the immune response [34].

### CIRCOs plot analysis and copy number variation analysis (CNV)

**Figure 4:**
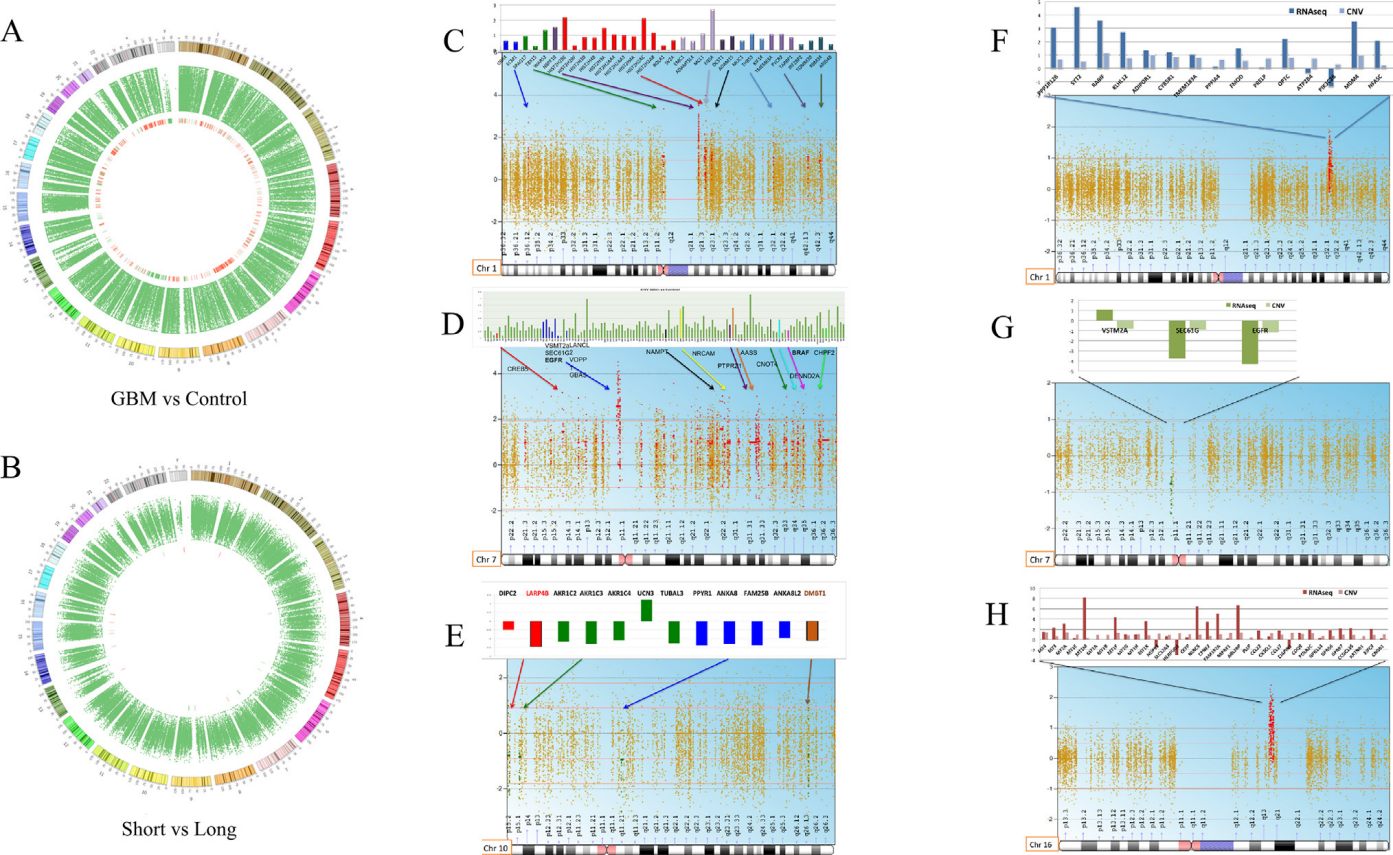
Whole CNV analysis of GBM vs. blood DNA control and S vs. L tumors (**A**) circos plot of all glioblastoma samples vs. control. (**B**) circos plot of Short vs. Long. (**C**–**E**)**,** chromosomes mostly affected by structural alterations deletion and amplifications comparing all GBM to a normal germinal blood DNA (chromosomes 1,7,10). Only the CNV data of each gene, localized in the amplified or deleted regions, is reported in the histograms. (**F**–**H**), chromosomes affected by deletions and amplifications that distinguish the S from the L group (chromosomes 1,7,16). The S and L comparison histograms report also the transcriptome data for each gene besides the CNV data.

A copy number variation analysis of all 13 GBM samples was performed against healthy blood DNA and reported in CIRCOs plots using a log2 ratio cut-off of 1 ± 0.5. The same analysis was conducted stratifying the GBM samples in the S and L group using a log2 ratio cut-off of 0.8 ± 0.5 SD (Figure 4A and 4B). We performed the analysis after a thorough analysis, and we decided to keep the parameter criteria very stringent, so to be able to filter the results and identify only relevant differences between groups in few chromosomes. All GBM samples had clear amplifications of several regions of chromosome 1 and 7 and mostly deletions in chromosome 10 as expected also from the literature (Figure 4C–4E). The differences were more settled between the S and L group, but it was possible to pinpoint some structural variations in chromosomes 1, 10 and 16 (Figure 4F–4H). For the GBM vs. healthy blood DNA comparison, we reported in the histograms only the CNV data of the genes localized in the amplified or deleted regions. For the comparison of the S and L groups, the CNV variation data of each gene is reported integrated with the transcriptome data as shown in Figure 4F–4H.

In Figure 4C–4E we report chromosomes 1, 7 and 10 mostly affected by deletions or amplifications comparing all GBM to a normal germinal blood DNA. For each chromosome, statistical significant genes involved in the chromosomal alteration are shown. Chromosome 1 carries several amplified genes already reported in cancer such as ADAMTSL4, Histon2H gene family, MUC1 and TOMM20 [35–39] (Figure 4C). Chromosome 7 is usually disrupted in many cancers, here we detected the amplification of several genes already implicated in glioblastoma such as: EGFR, SEPT14, NAMPT, NRCAM, AAS and PTPRZ1 [23, 40–44]. VSTM2A, LANCL2, SEC61G and VOPP1 were also amplified and are reported in the literature as fusion genes with EGFR in some glioblastoma patients [45, 46] (Figure 4D). The SEPT14 gene was also found fused to the EGFR gene in 4% of glioblastoma sensitive to EGFR inhibitors [42]. One gene noteworthy is the PTPRZ1, which is a receptor tyrosine phosphatase recently found upregulated in a fusion transcript with the MET gene and associated to glioma progression [47]. The Our transcriptome data revealed the PTPRZ1 gene also highly expressed in all GBM samples (Supplementary Table 1). Allelic loss on chromosome 10q is common to approximately 80% of glioblastoma cases, and it is not seen in lower grade astrocytoma. All genes, found located in the deleted chromosomal regions, had also no expression according to transcriptome data (Supplementary Table 1). As shown in Figure 4E, LARP4B and DMBT1 have already been reported as deleted in brain tumors. LARP4B, a RNA binding protein, is a candidate tumor suppressor gene in glioma. LARP4B expression was consistently decreased in human glioma stem cells and cell lines compared with normal neural stem cells. Overexpression of LARP4B in glioma cell lines strongly inhibited proliferation by inducing mitotic arrest and apoptosis [48]. DMBT1 (deleted in malignant brain tumor 1) is a putative tumor suppressor implicated in the carcinogenesis of medulloblastoma and glioblastoma [49, 50].

CNV analysis was also performed comparing the S versus the L group keeping statistical criteria very stringent in order to identify strong differences. We report here chromosome 1, 7 and 16, as mostly differentially altered between the S and L group (Figure 4F–4H). For each chromosome, we could integrate the transcriptome data obtained from the RNAseq analysis of the same patients as shown in the histograms (Figure 4F–4H). In Chromosome 1 (Figure 4F) the S group shows higher amplification of a series of genes compared to the L group, which are confirmed also at the transcriptome level, except for the PIK3C2B. For many genes, their function in cancer is still unknown while some others have already been implicated in cancer such as: RABIF, KLHL12, ADIPOR1, CYBR5R1 and PRELP [51–55]. The FMOD gene is a novel biomarker for prostate cancer, but it was found also upregulated in glioblastoma [56]. We observed MDM4 amplification, which has been reported to be associated to the chromotripsis phenomenon in glioblastoma progression [57]. Chromosome 7 appears as subjected to a deletion comparing the S and L group, but the analysis has created an artefact since the deletion describes, indeed, an amplification occurring actually in both groups with a different degree of intensity as represented in Figure 4G. Chromosome 7 is, therefore, less amplified for VSTM2A, SEC61G and EGFR in the S group. Several studies have shown that EGFR amplification and EGFR protein overexpression have no effect on prognosis when patients of all ages are analysed together. EGFR amplification is associated with a worse prognosis among younger patients and with a more favourable prognosis among older patients (aged >45, >55, or >60 years, depending on study) [35, 37–39, 42]. EGFR amplification does not preclude an unusually long survival, as 26% of glioblastoma patients surviving longer than 3 years have glioblastoma with EGFR amplification as in the L group of this study [45].

The CNV analysis of chromosome 16 revealed a highly amplified region in the S group compared to the L group as shown in Figure 4H. The integrated transcriptome data of the genes located in the amplified region revealed the same trend except for three genes (NUP93, HERPUD1 and CIAPIN1). In the rest of the amplified genes, resulted by CNV analysis, we identified two gene families involved in glioblastoma such as MT1M and GPR56 [21, 58]. Thanks to the transcriptome data, we could confirm that the differences in MT1M and GPR56 gene amplification matched the gene expression level between the S and L groups. The MT1M gene was indeed highly expressed in the S group with a significant adjusted *p*-value of 0.02 (*p* < 0.00005) according to the unsupervised differential gene expression analysis described earlier in our study (Figure 3E). The MT1M gene has been described in the literature as associated with poor survival in glioblastoma [21]. Antagonists of GPR56 inhibited, via Rho pathway, cell migration in glioblastoma human cell lines and in neuronal progenitor cells [58].

## DISCUSSION

**Figure 5:**
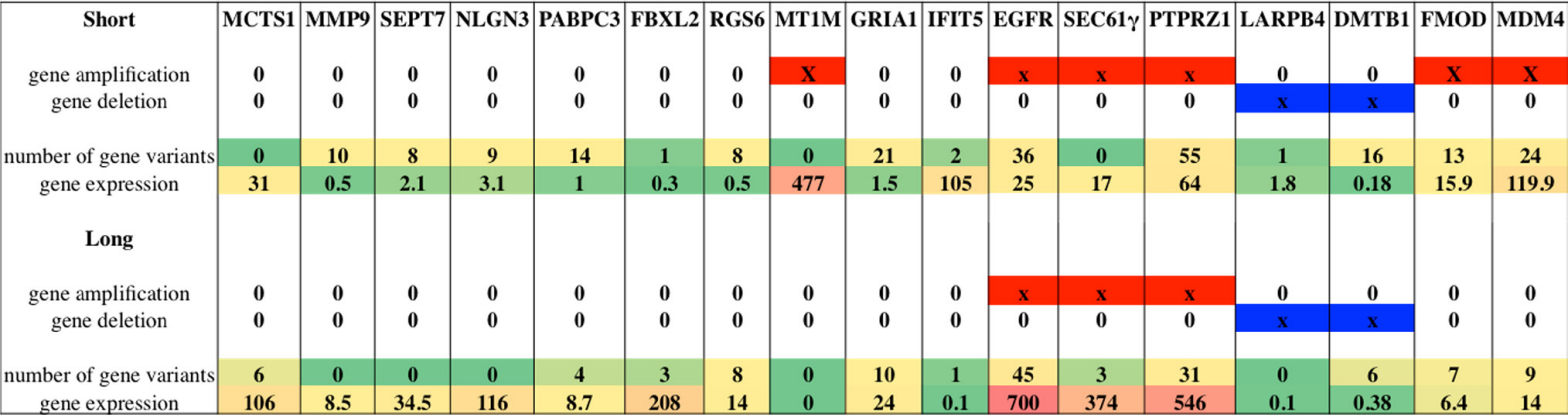
List of selected genes worthy of note after whole molecular analysis: genes where selected according to their relevance and involvement in glioblastoma For each gene it is provided information about gene amplification or deletion status, number of variants identified per group and gene expression value expressed in fragments per kilobase million (FPKM). Levels of expression are also shown with a green/yellow/red color scale. Red solid bars indicate gene amplification status, while blue solid bars indicate gene deletion status.

Despite the dramatic improvements in our understanding of glioblastoma fed by recent revolutions in molecular and systems biology, treatment advances for glioblastoma have progressed inadequately slowly. In this study having access to samples of GBM patients with exceptionally long survival (more than 25 months), we decided to compare their mutational and gene expression profile with a group of GBM patients with shorter RFS (Short: 6 months and Medium: 12 months). The particularity of this study is the extreme homogeneity of the patient casistics, which is made by all IDH1 wildtype primary glioblastoma. Subjects were chosen by the same pathologist to have same histology, similar condition and treatment. Patients underwent maximal tumor resection performed by the same surgeon at the University Hospital of Pisa, and treated by the same oncologist and radiotherapist. In fact, in this way we reduced the patients’ basic variability and enhance the pathological trait of interest (RFS). We mainly pointed at the extremes of the condition of interest (S tumors vs L tumors). The high homogeneity of our population allowed the identification of new molecular factors but also the confirmation of molecular factors identified before but never carried on and well framed in patients. Over all the exome data suggests that the S tumors might chronologically be in a more advanced state of the well-known mutation accumulation multi-step progression of cancer. L tumors have consistently a lower mutational rate. New players in this kind of cancer emerge from the whole analysis, confirmed at the RNA and DNA level, thus making it possible to establish potential oncodrivers or tumor suppressors elements (Figure 5). The unsupervised transcriptome analysis identifies new genes but also pinpoints the involvement of calcium and angiogenesis-related genes, metalloproteinases, ubiquitination factors and immune response genes. The less aggressive group of tumors (L) has, indeed, upregulation of the CBL, IL3, 5, GM CSF and dopamine degradation molecular signalling pathways that affect receptor-ligand binding and immune response. The application of these findings to patients’ clinical management is still remote even though many current experimental treatments are built on little biological comprehension. However, this study still provides an important foundation for further new thorough investigations on glioblastoma progression.

## METHODS

### Patient cohort and molecular analysis

13 human GBM subjects were selected from the archives of the Anatomy Pathology Institute of the University of Pisa, Italy. Subjects were chosen by the same pathologist, they have same histology, similar condition and treatment. All cases had a diagnosis of GBM with no previous history of any brain neoplasia. Patients underwent maximal tumor resection performed by the same surgeon at the University Hospital of Pisa, and all of them received external beam regional radiation of 60 Gy within 3–6 weeks after resection, together with TMZ treatment. Subjects were grouped depending on time of recurrence free survival (RFS) after first surgery: 6 Short (S) less than 6 months, 3 Medium (M) between 16 and 23 months and 4 Long (L) over 25 months (Table 1). This project was approved by the institutional review board of the University Hospital of Pisa (n. 3304/2011).

### Tumor samples

The selected GBM tumor tissues were formalin fixed and paraffin embedded (FFPE). For each sample, 2 sections of 10 µm thickness were cut using a new microtome blade for each tissue block and collected in a 1.5 ml tube. Samples were provided with their molecular characterization data for mutations in the isocitrate dehydrogenase 1 (IDH1) gene, amplification of the epidermal growth factor receptor (EGFR) gene, presence of the EGFR variantIII, and methylation of the promoter region of the O6-methylguanine-DNA methyltransferase (MGMT) gene (Table 1). The information regarding the size of the tumors and their localization within a certain brain region is also shown in Table 1.

### DNA and RNA extraction

DNA and RNA was extracted using the Maxwell^®^ 16 LEV DNA/RNA FFPE Purification Kit following exactly the manufacturer’s protocol.

### Next generation sequencing (NGS) analysis

A deep molecular characterization was performed on whole exome sequencing (WES) and whole transcriptome sequencing (WTS) with NGS technology (Ion Proton System, Thermo Fisher). WES was performed on 8 samples, 4 S and 4 L, while WTS was performed on all 13 samples (Table 1). Libraries were prepared after DNA/RNA amplification.

### Library preparation

#### WES

After DNA amplification (WGA4 – Sigma Aldrich, Saint Louis, MO, USA) libraries were prepared using the Ion TargetSeq Exome Enrichment kit for the Ion Proton System. Each fragment library was constructed from 1 μg of RNA-free genomic DNA.

#### WTS

cDNA retrotranscription was performed using SMARTer Universal Low Input RNA kit (Clontech Laboratories) that allows high-quality cDNA synthesis starting from as little as 200 pg of input RNA. cDNA libraries were prepared using the Ion TargetSeq Exome Enrichment kit, avoiding the fragmentation step was avoided.

### Template preparation and sequencing

To sequence our samples, we used Ion PI sequencing 200 kit (Life Technologies). The Ion PI Chip (Life Technologies) was prepared and calibrated for loading. We loaded the Ion PI Chip with our template-positive ISPs and we run on an Ion Proton sequencer.

### NGS data analysis

#### RNAseq

Bioinformatics analysis was carried out using several command line software included in Bio-Linux (http://nebc.nerc.ac.uk/tools/bio-linux/bio-linux-7-info) a custom version of Ubuntu 12.04 LTS. The unit of measurement used for reporting RNAseq expression values is FPKM, that stands for Fragments Per Kilobase of transcript per Million mapped reads. In RNAseq, the relative expression of a transcript is proportional to the number of cDNA fragments that originate from it. Heatmap plot was generated using the R package “cummeRbund”, a tool able to visualize data from cuffdiff output.

#### DNAseq

After Ion Proton run, data were automatically analyzed in Ion Torrent server, previously set for alignment to human genome (hg19 version). The variants were called by using Variant Caller Plugin, included in Torrent suite, by using high stringency parameters.

The .bam and .bai files made with Ion Torrent server were converted in mpileup format by using Samtools command and then used as input in CEQer software (CS) (www.ngsbicocca.org/html/ceqer.html), which is a tool for analyzing copy number variations (CNVs) and loss of heterozygosity (LOH). We performed the analysis by using a log2 ratio cut-off of 0.8 ± 0.5 SD to compare Short vs. Long and a cut-off of 1 ± 0.5 to compare GBM vs. a healthy blood DNA.

## SUPPLEMENTARY MATERIALS FIGURES AND TABLE




