# Supplementary material for: Cancer astrocytes have a more conserved molecular status in long recurrence free survival (RFS) IDH1 wild-type glioblastoma patients: new emerging cancer players
Source: Oncotarget. 2018 May 8;9(35):24014–27. doi: 10.18632/oncotarget.25265 (PMC5963624; doi:10.18632/oncotarget.25265)
Supplement: Supplementary file 1 [file oncotarget-09-24014-s001.pdf]

Cancer astrocytes have a more conserved molecular status in long recurrence free survival (RFS) IDH1 wild-type glioblastoma patients: new emerging cancer players

SUPPLEMENTARY MATERIALS

Sample	Group	Gene	Variant	Chr	Type	Genotype	Transcript	Consequence	CDS Position	Protein Position	Amino Acids	Codons	PolyPhen
M	Long	MCTS1	G>G/A	X	snv	het	NM_001137554.1	missense-variant	496	166	E/K	Gaa/Aaa	benign(0.305)
M	Long	MCTS1	C>C/G	X	snv	het	NM_001137554.1	missense-variant	523	175	L/V	Ctg/Gtg	probably damaging(0.985)
K	Long	MCTS1	G>G/A	X	snv	het	NM_001137554.1	splice-region-variant,intron-variant		0			
J	Long	MCTS1	G>G/A	X	snv	het	NM_001137554.1	missense-variant	172	58	E/K	Gaa/Aaa	benign(0.162)
L	Long	MCTS1	AG>AG/A	X	deletion	het	NM_001137554.1	frameshift-variant,truncation	251	84			
L	Long	MCTS1	G>G/A	X	snv	het	NM_001137554.1	missense-variant	181	61	E/K	Gaa/Aaa	probably damaging(0.913)
B	Short	SUPT16H	T>TG/TG	14	insertion	hom	NM_007192.3	frameshift-variant,elongation	1932-1933				
B	Short	SUPT16H	G>G/A	14	snv	het	NM_007192.3	splice-region-varian, intron-variant		0			
B	Short	SUPT16H	C>C/G	14	snv	het	NM_007192.3	splice-region-variant,synonymous-variant	1932	644	G	ggG/ggC	
A	Short	SUPT16H	T>T/A	14	snv	het	NM_007192.3	missense-variant	122	41	D/V	gAt/gTt	possibly damaging(0.754)
A	Short	SUPT16H	T>T/C	14	snv	het	NM_007192.3	missense-variant	1400	467	E/G	gAa/gGa	possibly damaging(0.842)
C	Short	SUPT16H	AT>AT/A	14	deletion	het	NM_007192.3	frameshift-variant, truncation	1054	352			
C	Short	SUPT16H	AG>AG/A	14	deletion	het	NM_007192.3	frameshift-variant, truncation	2470	824			
C	Short	SUPT16H	G>G/C	14	snv	het	NM_007192.3	splice-region-varian, intron-variant		0			
C	Short	SUPT16H	G>G/A	14	snv	het	NM_007192.3	missense-variant	1979	660	P/L	cCg/cTg	probably damaging(0.994)
C	Short	SUPT16H	C>C/T	14	snv	het	NM_007192.3	missense-variant	2438	813	S/N	aGt/aAt	probably damaging(0.927)
C	Short	SUPT16H	G>G/C	14	snv	het	NM_007192.3	missense-variant	2468	823	A/G	gCg/gGg	possibly damaging(0.552)
C	Short	SUPT16H	A>A/T	14	snv	het	NM_007192.3	missense-variant	2478	826	N/K	aaT/aaA	possibly damaging(0.773)
E	Short	SUPT16H	A>A/G	14	snv	het	NM_007192.3	missense-variant	2474	825	V/A	gTa/gCa	possibly damaging(0.811)
B	Short	BMPR2	GA>G/G	2	deletion	hom	NM_001204.6	frameshift-variant, truncation	1493	498			
B	Short	BMPR2	G>G/C	2	snv	het	NM_001204.6	missense-variant	403	135	D/H	Gac/Cac	possibly damaging(0.53)
A	Short	BMPR2	TAG>TAG/T	2	deletion	het	NM_001204.6	frameshift-variant, truncation	2434-2435	812			
A	Short	BMPR2	CTG>CTG/C	2	deletion	het	NM_001204.6	frameshift-variant, truncation	402-403				
A	Short	BMPR2	A>A/G	2	snv	het	NM_001204.6	splice-region-variant, synonymous-variant	249	83	G	ggA/ggG	
A	Short	BMPR2	G>G/C	2	snv	het	NM_001204.6	missense-variant	368	123	C/S	tGt/tCt	probably damaging(0.999)
A	Short	BMPR2	G>G/A	2	snv	het	NM_001204.6	missense-variant	1100	367	G/E	gGg/gAg	probably damaging(0.958)
A	Short	BMPR2	T>A/C	2	snv	het	NM_001204.6	missense-variant	2959	987	S/P	Tcc/Ccc	possibly damaging(0.659)
C	Short	BMPR2	C>G/T	2	snv	het	NM_001204.6	missense-variant	1505	502	A/V	gCt/gTt	possibly damaging(0.619)
C	Short	BMPR2	C>T/G	2	snv	het	NM_001204.6	missense-variant	1505	502	A/G	gCt/gGt	possibly damaging(0.666)
E	Short	BMPR2	C>C/T	2	snv	het	NM_001204.6	missense-variant	383	128	T/I	aCt/aTt	probably damaging(0.999)
E	Short	BMPR2	G>G/A	2	snv	het	NM_001204.6	missense-variant	1492	498	E/K	Gag/Aag	probably damaging(0.999)
B	Short	C2orf66	GCA>GCA/G	2	deletion	het	NM_213608.2	frameshift-variant, truncation	71-72	24			
B	Short	C2orf66	C>C/T	2	snv	het	NM_213608.2	missense-variant	161	54	R/K	aGa/aAa	probably damaging(0.952)
A	Short	C2orf66	TC>TC/T	2	deletion	het	NM_213608.2	frameshift-variant, truncation	161	54			
A	Short	C2orf66	T>T/A	2	snv	het	NM_213608.2	missense-variant	60	20	R/S	agA/agT	benign(0.045)
A	Short	C2orf66	T>T/A	2	snv	het	NM_213608.2	missense-variant	143	48	K/M	aAg/aTg	probably damaging(0.997)
A	Short	C2orf66	A>A/G	2	snv	het	NM_213608.2	missense-variant	149	50	L/P	cTc/cCc	probably damaging(0.997)
C	Short	C2orf66	G>G/A	2	snv	het	NM_213608.2	missense-variant	50	17	T/I	aCc/aTc	probably damaging(0.994)
E	Short	C2orf66	A>A/T	2	snv	het	NM_213608.2	missense-variant	149	50	L/H	cTc/cAc	probably damaging(0.997)
B	Short	MMP9	CG>CA/C	20	deletion	het	NM_004994.2	frameshift-variant, truncation	1395	465			
B	Short	MMP9	G>G/A	20	snv	het	NM_004994.2	missense-variant	1403	468	C/Y	tGc/tAc	possibly damaging(0.487)
A	Short	MMP9	GC>GC/G	20	deletion	het	NM_004994.2	frameshift-variant, truncation	2022	674			
A	Short	MMP9	A>A/C	20	snv	het	NM_004994.2	missense-variant	1363	455	T/P	Acc/Ccc	possibly damaging(0.544)
A	Short	MMP9	G>A/A	20	snv	hom	NM_004994.2	missense-variant, splice-region-variant	2003	668	R/Q	cGa/cAa	benign(0.179)
C	Short	MMP9	GC>GC/G	20	deletion	het	NM_004994.2	frameshift-variant, truncation	2022	674			
C	Short	MMP9	C>C/CTT	20	insertion	het	NM_004994.2	frameshift-variant, elongation	1406-1407	469			
C	Short	MMP9	C>C/T	20	snv	het	NM_004994.2	missense-variant	902	301	A/V	gCc/gTc	benign(0.013)
C	Short	MMP9	G>G/A	20	snv	het	NM_004994.2	missense-variant	1403	468	C/Y	tGc/tAc	possibly damaging(0.487)
E	Short	MMP9	AC>AC/A	20	deletion	het	NM_004994.2	frameshift-variant,truncation	2028	676			
B	Short	NLGN3	TG>TT/T	X	deletion	het	NM_181303.1	frameshift-variant, truncation	1765	589			
B	Short	NLGN3	G>G/GTGTT	X	insertion	het	NM_181303.1	frameshift-variant, elongation	1240-1241	414			
B	Short	NLGN3	A>T/T	X	snv	hom	NM_181303.1	missense-variant	2044	682	N/Y	Aat/Tat	benign(0.166)
B	Short	NLGN3	TG>T/TT	X	snv	het	NM_181303.1	stop-gained	1764-1765		FE/F*	ttTGag/ ttTTag	
A	Short	NLGN3	TG>TG/T	X	deletion	het	NM_181303.1	frameshift-variant, truncation	1594	532			
A	Short	NLGN3	C>C/T	X	snv	het	NM_181303.1	stop-gained	1801	601	Q/*	Cag/Tag	
A	Short	NLGN3	C>C/T	X	snv	het	NM_181303.1	missense-variant	2518	840	P/S	Ccc/Tcc	probably damaging(0.982)
C	Short	NLGN3	TG>T/T	X	deletion	hom	NM_181303.1	splice-region-variant- intron-variant truncation		0			
E	Short	NLGN3	TC>TC/T	X	deletion	het	NM_181303.1	frameshift-variant,truncation	1722	574			
B	Short	DUSP12	CGTTT>C/C	1	deletion	hom	NM_007240.1	frameshift-variant,truncation	625-628				
B	Short	DUSP12	G>G/A	1	snv	het	NM_007240.1	missense-variant	367	123	V/M	Gtg/Atg	probably damaging(0.993)
A	Short	DUSP12	GT>GT/G	1	deletion	het	NM_007240.1	frameshift-variant, truncation	995	332			
A	Short	DUSP12	G>G/T	1	snv	het	NM_007240.1	stop-gained	1000	334	G/*	Gga/Tga	
C	Short	DUSP12	G>G/A	1	snv	het	NM_007240.1	missense-variant	911	304	G/D	gGt/gAt	probably damaging(0.998)
E	Short	DUSP12	A>A/G	1	snv	het	NM_007240.1	missense-variant	931	311	R/G	Agg/Ggg	possibly damaging(0.826)
B	Short	RPF2	GC>G/G	6	deletion	hom	NM_032194.1	frameshift-variant, truncation	441	147			
B	Short	RPF2	G>GA/GA	6	insertion	hom	NM_032194.1	frameshift-variant, elongation	442-443	148			
B	Short	RPF2	C>C/A	6	snv	het	NM_032194.1	missense-variant	508	170	P/T	Ccc/Acc	possibly damaging(0.587)
A	Short	RPF2	GA>GA/G	6	deletion	het	NM_032194.1	frameshift-variant, truncation	504	168			
C	Short	RPF2	G>G/A	6	snv	het	NM_032194.1	missense-variant	646	216	E/K	Gag/Aag	possibly damaging(0.721)
E	Short	RPF2	G>G/T	6	snv	het	NM_032194.1	missense-variant	750	250	K/N	aaG/aaT	probably damaging(0.959)
B	Short	SEPT7	TA>TA/T	7	deletion	het	NM_001788.5	frameshift-variant, truncation	747	249			
B	Short	SEPT7	G>G/GT	7	insertion	het	NM_001788.5	frameshift-variant, elongation	748-749	250			
A	Short	SEPT7	TCC>TCC/T	7	deletion	het	NM_001788.5	frameshift-variant, truncation	232-233	78			
A	Short	SEPT7	A>A/G	7	snv	het	NM_001788.5	splice-acceptor-variant		0			
C	Short	SEPT7	TA>TG/T	7	deletion	het	NM_001788.5	splice-acceptor-variant, truncation		0			
C	Short	SEPT7	G>G/A	7	snv	het	NM_001788.5	missense-variant, splice-region-variant	724	242	D/N	Gac/Aac	
C	Short	SEPT7	TA>T/TG	7	snv	het	NM_001788.5	splice-acceptor-variant, intron-variant		0			
E	Short	SEPT7	GCA>GCA/G	7	deletion	het	NM_001788.5	frameshift-variant, splice-region-variant, truncation	67-68	23			

Supplementary Figure 1: Variants type and details distributed through each sample in the S and L group. PolyPhen tool is used to estimate possible consequences on protein structure. Letters from A to M represent the label of each sample (one color for each sample).

gene	locus	sample_1	sample_2	value_1	value_2	q_value/FDR
**OCM**	chr7:5920428-5925994	Medium	Long	**0**	**30.4905**	0.0223628
**FAM184A**	chr6:119280995-119470358	Medium	Long	**0.106381**	**99.7196**	0.0384849
**FOXP1**	chr3:71003864-71633140	Medium	Long	**0.446157**	**22.3871**	0.0223628
**ZMYND11**	chr10:180404-300577	Medium	Long	**0.504517**	**90.9134**	0.032448
**ACE**	chr17:61554421-61575741	Medium	Long	**0.795322**	**148.925**	0.0481061
**GUCD1**	chr22:24936390-24970932	Medium	Long	**1.02987**	**192.284**	0.0481061
**SLC6A9**	chr1:44457279-44497164	Medium	Long	**1.24926**	**129.964**	0.0223628
**PAQR6**	chr1:156182778-156217908	Medium	Long	**1.27094**	**382.889**	0.0223628
**TCF7L2**	chr10:114710008-114927436	Medium	Long	**1.6023**	**119.056**	0.0223628
**DMD**	chrX:31137344-33357726	Medium	Long	**1.73469**	**291.081**	0.0223628
**C3orf17**	chr3:112721291-112738555	Medium	Long	**1.85436**	**130.523**	0.045651
**LMNA**	chr1:156052336-156109880	Medium	Long	**3.13916**	**175.146**	0.0223628
**SORT1**	chr1:109852187-109940563	Medium	Long	**4.59542**	**156.114**	0.0481061
**EIF4G3**	chr1:21132784-21503381	Medium	Long	**5.3661**	**294.813**	0.0384849
**NBPF11**	chr1:147574322-149109725	Medium	Long	**5.57296**	**97.3059**	0.0223628
**ATXN7**	chr3:63850232-63989136	Medium	Long	**15.945**	**0.236583**	0.0384849
**ATXN3**	chr14:92524895-92572965	Medium	Long	**45.228**	**0.247906**	0.0384849
**TRO**	chrX:54946995-54957866	Medium	Long	**75.0432**	**0.34921**	0.045651
**SNORD115-25**	chr15:25451408-25477615	Medium	Long	**444.847**	**0**	0.0384849
**WDR4**	chr21:44263189-44299699	Short	Medium	**0.846406**	**60.57**	0.0384849
**ARHGAP8**	chr22:45064426-45258664	Short	Medium	**0.891028**	**27.971**	0.0384849
**MAP7**	chr6:136663418-136871957	Short	Medium	**6.25896**	**0.354966**	0.045651
**AMT,NICN1**	chr3:49454210-49466757	Short	Medium	**7.95951**	**0.192892**	0.0223628
**RAB27A**	chr15:55495163-55582013	Short	Medium	**11.2309**	**0.147277**	0.045651
**TTN**	chr2:179059207-179672150	Short	Medium	**12.0656**	**0.526317**	0.0223628
**ATG5**	chr6:106632351-106773695	Short	Medium	**19.9251**	**0.1946**	0.0223628
**BCAR1**	chr16:75262927-75301951	Short	Medium	**20.1569**	**0.427328**	0.0223628
**ZNF410**	chr14:74353317-74398991	Short	Medium	**20.9481**	**0.561508**	0.045651
**LDB3**	chr10:88428205-88495824	Short	Medium	**29.2972**	**0.312961**	0.0223628
**MID2**	chrX:107069083-107174867	Short	Medium	**32.3853**	**0.184674**	0.0223628
**NFX1**	chr9:33290417-33371155	Short	Medium	**37.6796**	**0.803842**	0.032448
**LOC645638**	chr17:58160926-58165828	Short	Medium	**41.7757**	**0**	0.0481061
**PLEKHA5**	chr12:19282625-19529333	Short	Medium	**43.1029**	**0.578009**	0.0223628
**DMD**	chrX:31137344-33357726	Short	Medium	**45.8031**	**1.73469**	0.032448
**EXD2**	chr14:69658193-69710737	Short	Medium	**60.9554**	**0.633493**	0.032448
**ARMC8**	chr3:137906089-138048728	Short	Medium	**68.2671**	**1.6873**	0.0223628
**GIGYF2**	chr2:233562014-233725289	Short	Medium	**79.793**	**0.806054**	0.0223628
**FAM178A**	chr10:102672325-102724891	Short	Medium	**87.9994**	**1.66979**	0.045651
**NIN**	chr14:51186480-51297839	Short	Medium	**97.8301**	**2.57399**	0.0481061
**MSL3**	chrX:11776277-11793872	Short	Medium	**99.4702**	**0.77299**	0.0223628
**C17orf62**	chr17:80347085-80408707	Short	Medium	**111.257**	**1.07858**	0.0223628
**HDLBP**	chr2:242166681-242293441	Short	Medium	**114.665**	**1.89424**	0.0223628
**MYLK**	chr3:123304402-123603149	Short	Medium	**116.471**	**1.17297**	0.0223628
**ITPK1**	chr14:93403258-93582263	Short	Medium	**139.858**	**2.04783**	0.032448
**TSC22D1**	chr13:45006278-45154568	Short	Medium	**140.928**	**3.42618**	0.0223628
**SON**	chr21:34915343-34949820	Short	Medium	**494.305**	**21.8508**	0.0481061
**C17orf76-AS1**	chr17:16342300-16395505	Short	Medium	**662.382**	**5.63239**	0.0223628

Supplementary Figure 2: 48 DEG in the M group compared to the S and L group. The DMD gene (highlighted in yellow) is the only gene found that is statistically different from both the S and L group. RNAseq expression value levels are reported in fragments per kilobase million (FPKM). Levels of expression are also shown with a green/yellow/red color scale of the differentially expressed genes (DEG): dark red indicates the highest expression level and dark green the lowest.

Supplementary Table 1: Whole differential gene expression analysis performed comparing the S, M and L groups.
See Supplementary_Table_1
